# Regional cortical thinning in young adults with schizophrenia but not psychotic or non-psychotic bipolar I disorder

**DOI:** 10.1186/s40345-018-0124-x

**Published:** 2018-07-11

**Authors:** Douglass Godwin, Kathryn I. Alpert, Lei Wang, Daniel Mamah

**Affiliations:** 1 Department of Psychiatry, Washington University Medical School, St. Louis, USA; 2 Department of Psychiatry and Behavioral Sciences, Northwestern University Feinberg School of Medicine, Chicago, USA

**Keywords:** Bipolar disorder, Schizophrenia, Cortical thickness, MRI

## Abstract

**Background:**

Schizophrenia shares some genetic risk and clinical symptoms with bipolar disorder. Clinical heterogeneity across subjects is thought to contribute to variable structural imaging findings across studies. The current study investigates cortical thickness in young adults diagnosed with schizophrenia or bipolar I disorder with a history of hyperthymic mania. We hypothesize that cortical thickness will be most similar between SCZ and the psychotic bipolar 1 disorder subtype.

**Methods:**

Patients with schizophrenia (n = 52), psychotic bipolar I disorder (PBD; n = 49) and non-psychotic bipolar I disorder (NPBD; n = 24) and healthy controls (n = 40) were scanned in a 3T Trio MRI. The thickness of 34 cortical regions was estimated with FreeSurfer, and analyzed using univariate analyses of variance. Relationships to psychotic (SAPS) and negative (SANS) symptoms were investigated using linear regression.

**Results:**

Cortical thickness showed significant group effects, after covarying for sex, age, and intracranial volume (p = 0.001). SCZ subjects had thinner paracentral, inferior parietal, supramarginal and fusiform cortices compared to CON. Caudal anterior cingulate cortical thickness was increased in SCZ, PBD and NPBD. Cortical thickness in PBD and NPBD were not significantly different from controls. Significant partial correlations were observed for SAPS severity with middle temporal (r = − 0.26; p = 0.001) and fusiform (− 0.26; p = 0.001) cortical thickness.

**Conclusions:**

Individuals with SCZ displayed significantly reduced cortical thickness in several cortical regions compared to both CON and bipolar. We found that SCZ participants had significant cortical thinning relative to CON and bipolar disorder most significantly in the frontal (i.e. paracentral), parietal (i.e. inferior parietal, supramarginal), and temporal (i.e. middle temporal, fusiform) cortices.

## Background

Bipolar disorder has substantial genetic and familiar overlap with schizophrenia and often share clinical features, including the presence of psychotic symptoms (Cardno and Owen 2014; Moskvina et al. 2009; Mamah and Barch 2011; Fischer and Carpenter 2009). Over 60% of bipolar disorder patients have had a lifetime history of at least one psychotic symptom (Keck et al. 2003; Coryell et al. 2001; Goodwin and Jamison 2007). Some studies have also found that schizophrenia susceptibility genes are more commonly shared with psychotic bipolar patients, compared to bipolar patients without a psychotic disorder history (Moskvina et al. 2009; Ivleva et al. 2008, 2010; Green et al. 2005; Potash 2006; Tamminga et al. 2013).

There has been continued interest in the use of neuroimaging to investigate intermediate phenotypes common across schizophrenia and bipolar disorder. The cortical structure of the brain is commonly quantitatively measured via gray matter volume, a measurement that is dependent on both cortical thickness and surface area of a region. These structural measures are thought to be heritable (Panizzon et al. 2009) and can therefore provide insight into the genetic differences between patient populations. However, the surface area and cortical thickness appear to be genetically uncorrelated (Panizzon et al. 2009). Thus, volume measurements, which combine aspects of both traits, are likely influenced by some combination of these genetic factors. Cortical thickness is thought to be dependent on the size, number, and arrangement of neurons and neuroglia in the cortex (Hatton et al. 2013; Goldman-Rakic 1988; Kuperberg et al. 2003; Rakic and Caviness 1995; Winkler et al. 2010). As such, cortical thinning relative to normal populations has implications about the preservation of, or lack thereof, network architecture in the cortex. To the extent abnormal function in cortical and subcortical networks, which are thought to be perturbed in both diseases (Uhlhaas 2013; Hulshoff Pol et al. 2012; Menon 2011), can be explained by abnormality of structure, the region-specific nature of cortical thinning could provide insight into differential developmental trajectories of brain disorders (Fischl et al. 1999; Fischl and Dale 2000).

Several brain studies investigating cortical structure have been conducted in schizophrenia and bipolar disorder patients. A meta-analysis of voxel-based morphometric studies in schizophrenia and bipolar disorder identified overlap of gray matter deficits in paralimbic cortical regions (including the anterior cingulate and insula) across the two diagnoses, with schizophrenia patients showing more extensive cortical deficits, also including neocortical and limbic regions (Ellison-Wright and Bullmore 2010). A recent large meta-analysis by the ENIGMA schizophrenia working group found widespread cortical thinning in schizophrenia, as well as reduced surface area (van Erp et al. 2017). Similarly, Rimol et al. (2012) found widespread cortical thinning and circumscribed cortical area reductions in schizophrenia patients, but not in bipolar disorder patients. However, the largest study of cortical thickness and surface area in bipolar disorder was conducted within the ENIGMA Consortium, and involved 2447 adult bipolar disorder patients (Hibar et al. 2018). These authors found cortical thinning in frontal, temporal and parietal regions, as well as an association of reduced cortical surface area with psychosis history (Hibar et al. 2018).

Heterogeneity in patient populations studied can influence the structural imaging findings observed (Zhang et al. 2015). Thus, studying more homogenous populations may provide greater understanding of cortical thickness in disease. For example, investigating a wide age range of subjects can introduce confounds unrelated to the illness and associated with older age. Studying cortical thickness in younger and older bipolar disorder populations separately, have provided additional insights into the disease, showing substantial cortical thinning only in the older bipolar patients (Hibar et al. 2018). Studying the clinical subtypes separately, can also be valuable. Patients with bipolar I disorder and bipolar II disorder are sometimes pooled as one group, despite the former representing a subtype with longer duration clinical episodes (“mania”) than the latter (“hypomania”) (American_Psychiatric_Association 2013). Significant reductions in cortical volume and thickness however has been reported by some authors to be limited to bipolar I disorder patients (Maller et al. 2014; Abe et al. 2016; Rimol et al. 2010). Finally, separating bipolar patients based on psychotic disorder histories often show differential effects, usually with the psychotic bipolar patients showing structural changes more closely related to that of schizophrenia patients (Strasser et al. 2005; Mamah et al. 2016; Womer et al. 2014).

The current study compares cortical thickness between young adult (aged 18–30 years) control subjects and patients with schizophrenia, psychotic bipolar I disorder and non-psychotic bipolar I disorder. Bipolar disorder subjects all had a history of hyperthymic manic episodes, while patients with a history of exclusively irritable manic episodes were excluded. In addition, none of our participants met criteria for a substance use disorder in the past 6 months of assessment, to minimize substance related effects on cortical thickness. We hypothesized that patterns of cortical thinning in schizophrenia patients will share some similarity with psychotic but not non-psychotic bipolar disorder patients.

## Methods

### Participants

All participants provided written informed consent and experimental procedures were approved by the Washington University School of Medicine institutional review board. Four diagnostic groups were included in the current study: healthy controls (CON; n = 40), schizophrenia (SCZ, n = 52), psychotic bipolar I disorder (PBD, n = 49) and non-psychotic bipolar I disorder (NPBD, n = 24). Participant groups were diagnosed on the basis of a consensus between a research psychiatrist and a trained research assistant who used the Structured Clinical Interview for DSM-IV Axis I Disorder (First et al. 2002; Lobbestael et al. 2011). CON subjects were required to have no lifetime history of psychotic or mood disorders. Bipolar (BD) participant patients were required to meet DSM-IV criteria for Bipolar I Disorder and were classified as psychotic BD if the participant had a psychotic event over the course of their lifetime, as assessed via the Structured Clinical Interview for the DSM (SCID). All psychotic events were reported to have occurred during manic episodes in bipolar participants. Participants were excluded if they: (a) met DSM-IV criteria for substance dependence or severe/moderate abuse during the prior 6 months; (b) had a clinically unstable or severe general medical disorder; or (c) had a history of head injury with documented neurological sequelae or loss of consciousness. Additionally, to minimize clinical heterogeneity within the BD group, only participants with a history of euphoric mania (versus mania characterized by primarily irritable mood) were included in the study.

Table 1 shows demographic data across the four participant groups. All patients were on stable medication for at least 4 weeks prior to assessment. Based on the results of the Young Mania Rating Scale (YMRS), the majority of bipolar participants were euthymic at the time of admission into the study. Four participants (3 PBD, 1 NPBD) scored between 13 and 20 suggesting a potential case of hyper- or hypomania. Three participants (2 PBD, 1 NPBD) scored above 20 indicating a probable case of hyper- or hypomania (Young et al. 1978; Marchand et al. 2005). The YMRS was only collected for bipolar patients. The average duration of illness for the psychotic bipolar group was 7.87 years while the average duration of illness for the non-psychotic group was 8.58 years.

**Table 1:** Demographics and Clinical Information

Characteristics	CON (n = 40)	SCZ (n = 52)	PBD (n = 49)	NPBD (n = 24)
Mean age (SD)	24.9 (5.0)	26.1 (4.1)	25.3 (3.7)	26.2 (3.7)
Sex (%)				
Male	20 (50.0)	38 (73.1)	20 (40.8)	8 (33.3)
Female	20 (50.0)	14 (26.9)	29 (59.2)	16 (66.7)
Race (%)				
Asian	2 (5.0)	0	1 (2.0)	2 (8.3)
Black	20 (50.0)	27 (51.9)	13 (26.5)	2 (8.3)
Hispanic	0	0	3 (6.1)	0
White	18 (45.0)	25 (48.1)	30 (61.2)	18 (75.0)
Multiracial	0	0	2 (4.1)	2 (8.3)
Handedness (%)				
Left	4 (10.0)	2 (3.8)	4 (8.2)	3 (12.5)
Right	36 (90.0)	50 (96.2)	45 (91.8)	21 (87.5)
History of use disorder (%)^a^				
Alcohol	4 (10.0)	17 (32.7)	23 (46.9)	12 (50.0)
Cannabis	0	21 (40.4)	17 (34.7)	6 (25.0)
Stimulant	1 (2.5)	1 (1.9)	1 (2.0)	0
Opioid	0	1 (1.9)	2 (4.1)	0
Cocaine	0	0	6 (12.2)	1 (4.2)
Hallucinogen	0	0	2 (4.1)	1 (4.2)
Psychotropic medication (%)				
Typical antipsychotic	0	8 (13.6)	3 (6.1)	0
Atypical antipsychotic	0	37 (72.5)	30 (62.2)	6 (25.0)
SSRI	0	15 (29.4)	24 (49.0)	10 (41.7)
Other antidepressants^b^	0	3 (5.8)	12 (24.5)	7 (29.2)
Stimulant	0	0	4 (8.2)	3 (12.5)
Mood stabilizer	0	11 (21.6)	33 (67.3)	18 (75.0)
Benzodiazepines	0	1 (2.0)	20 (40.8)	6 (25.0)
Anticholinergic	0	0	8 (16.3)	2 (8.3)
None	40 (100.0)	13 (25.5)	6 (12.2)	1 (4.2)
Symptom domains (SD)				
SAPS^c^	0.25 (.2)	3.56 (3.0)	1.35 (1.9)	0.42 (0.8)
Hallucination subscale	0	1.40 (1.8)	0.39 (0.9)	0
Delusion subscale	0.05 (.221)	2.12 (1.4)	0.96 (1.3)	0.42 (0.8)
SANS^d^	2.30 (2.3)	10.25 (3.9)	3.18 (3.8)	3.42 (2.4)
Flat Affect subscale	0.08 (0.4)	1.85 (1.3)	0.59 (0.9)	0.71 (1.2)
Alogia subscale	0.05 (0.3)	1.23 (1.8)	0.16 (0.5)	0.13 (0.4)
Anhedonia subscale	0.38 (0.8)	2.48 (1.1)	1.31 (1.4)	1.04 (1.1)
Amotivation subscale	0.73 (1.1)	3.02 (1.0)	1.24 (1.4)	0.92 (1.2)
Attention subscale	1.08 (1.3)	1.67 (1.3)	0.88 (1.1)	0.63 (1.0)

^a^Other than for nicotine use disorder, participants did not meet criteria for a use disorder in the last 6 months

^b^Refers to antidepressants other than selective serotonin reuptake inhibitors (SSRI)

^c^Maximum possible score on the Structured Assessment of Positive Symptoms (SAPS) is 16

^d^Maximum possible score on the Structured Assessment of Negative Symptoms (SANS) is 20

### Clinical assessment

Symptoms were assessed using the Scale for the Assessment of Negative Symptoms (SANS) and the Scale for the Assessment of Positive Symptoms (SAPS). Specific subscale scores were summed to derive measures of positive symptoms (i.e. hallucination, delusion) and negative symptoms (i.e. flat affect, alogia, anhedonia, attention and amotivation subscales). Disorganized symptoms were not included in SAPS scores so as to only reflect the presence of positive symptoms.

### Image and cortical thickness acquisition

Magnetic resonance (MR) scans were obtained using a Siemens (Erlangen, Germany) 3T Tim TRIO Scanner at Washington University Medical School. T1-weighted images were acquired using a sagittal MPRAGE 3D sequence (TR = 2400 ms, TE = 3.16 ms, flip = 8°; voxel size = 1 × 1 × 1 mm).

Cortical thickness of 34 brain cortical regions for each hemisphere were obtained using FreeSurfer version 5.1.0 software (Fischl et al. 1999; Dale et al. 1999). Gray-matter (GM) and white-matter (WM) boundaries were reconstructed in order to estimate the distance between those surfaces across the cortex in order to calculate the cortical thickness (Dale et al. 1999; Han et al. 2006; Rosas et al. 2002). Our preprocessing included automated skull stripping, removal of subcortical surfaces, transformation into Talairach space, and a spatial smoothing algorithm (Dale et al. 1999). Measurements of intracranial volume (ICV) were determined via the automated Freesurfer pipeline. These surfaces were then inflated into spheres in order to be registered to a common space via an automated procedure based on identification of cortical features identified in a predefined atlas (Fischl et al. 1999). Thickness for each region of interest (ROI) was calculated on a per-vertex basis based on the distance between closest points between gray and white-matter surfaces and then averaged across vertices within each region of interest (Fischl et al. 1999; Dale et al. 1999).

### Statistical analysis

Statistical analyses were performed using IBM’s SPSS software (IBM SPSS Statistics for Macintosh, Version 24.0, Armonk, NY). We examined group differences in SAPS and SANS measures using a multivariate analysis of covariance, covarying for age and sex. Differences in cortical thickness between subjects of different diagnostic groups were initially tested using a repeated measures analysis of covariance, covaried for age, sex, and ICV, treating hemisphere and region as repeated-measures. ICV was used here as a covariate given past evidence of cranial size differences in individuals with schizophrenia (Ward et al. 1996). This repeated-measures test revealed no main effect of hemisphere. Thus, subsequent testing was performed on cortical thickness values averaged across hemisphere in order to reduce the number tests for multiple comparisons considerations. We then tested for univariate effects of diagnostic group on cortical thickness within each region, with post hoc analyses of pairwise differences in groups in regions showing a univariate effect. To assess the relationship between clinical variables and cortical thickness, we performed a set of linear regressions to predict SAPS and SANS scores for each ROI. Included in these models were predictors for age, sex, ICV as well as dummy predictors for SCZ, PBD, and NPBD group membership in addition to a cortical thickness regressor. Given the number of regions tested, multiple comparisons corrections are noted when performed.

## Results

### Clinical measures

**Fig. 1:**
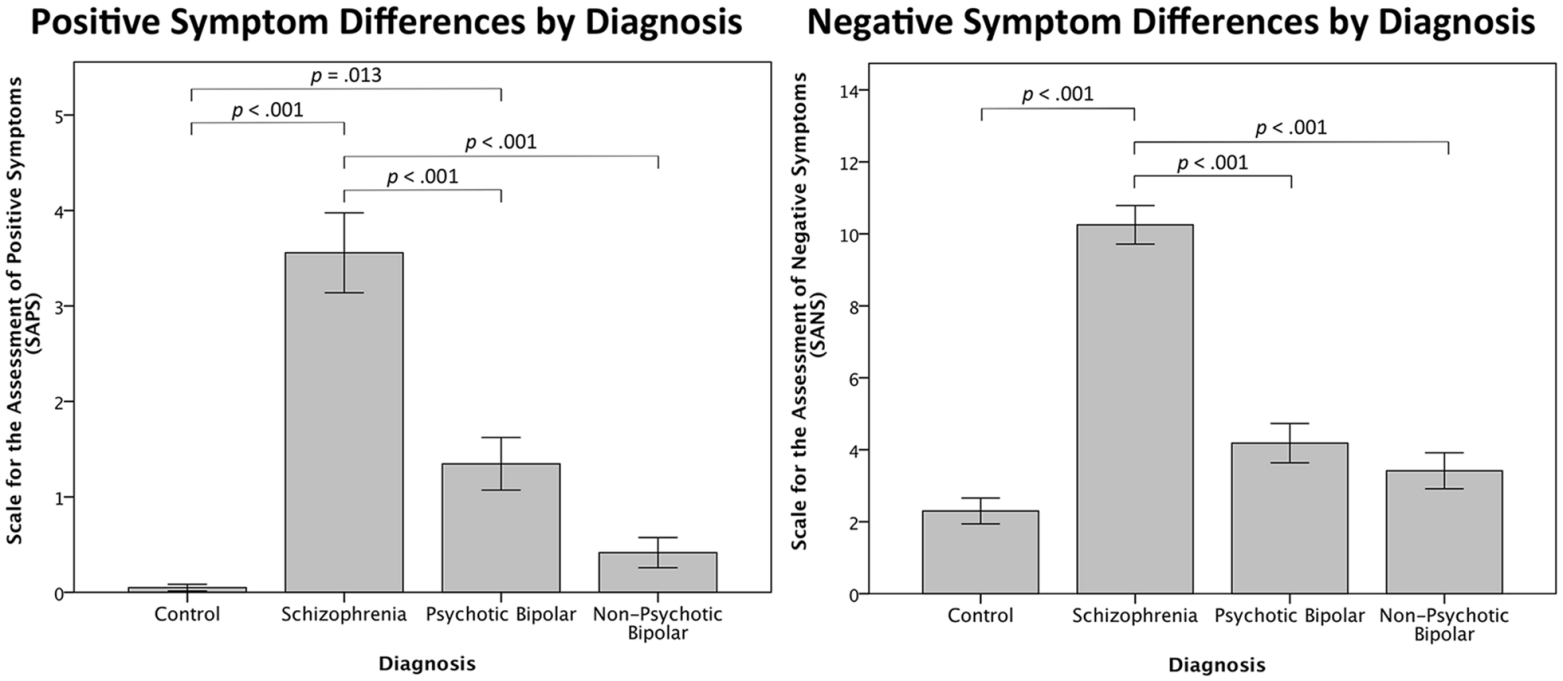
Clinical measures of positive and negative symptoms by diagnosis. (left) Relationship between positive symptoms by diagnosis as assessed by SAPS. (right) Negative symptom scores as assessed by SANS by diagnosis. Significant pairwise comparisons shown in both figures, Bonferroni corrected for multiple comparisons. Error bars represent within-group standard error

Based on multivariate testing, there was a significant main effect of diagnosis (F_(6,310)_ = 20.530, Wilk’s Λ = 0.512, *p* < 0.001, η^2^ = 0.284) after controlling for age and sex, indicating a difference in SAPS and SANS scores between groups. Differences in scores were driven by higher ratings in the SCZ and PBD groups relative to controls (CON) in the SAPS data, and SCZ relative to all other groups in the SANS scores (Table 1; Fig. 1).

### Group analysis of cortical thickness

**Fig. 2:**
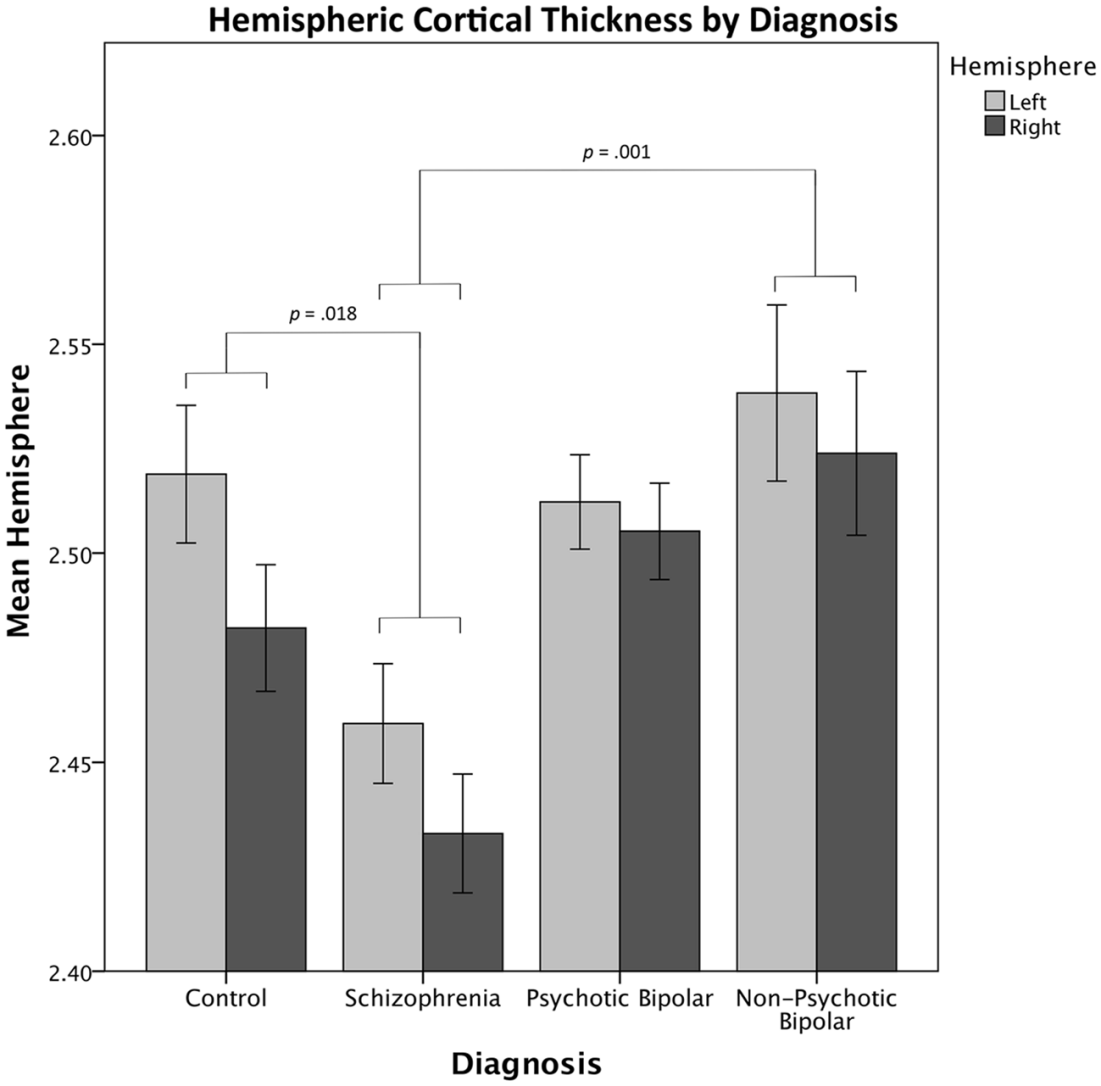
Average cortical thickness per hemisphere by diagnosis. In repeated-measures testing, a significant main effect of diagnosis was observed, with no significant main effect of hemisphere. Significance comparisons represent pairwise contrasts between diagnostic groups. Significance values greater than 0.05 not shown. Pairwise comparisons were Bonferroni corrected for multiple comparisons. Error bars represent within-group standard error

Covarying for sex, age, and ICV, a repeated-measures test of cortical thickness showed a significant main effect of diagnosis (F_(3,158)_ = 5.664, *p* = 0.001, η^2^ = 0.058; Fig. 2) and region (F_(33,514)_ = 23.074, *p* < 0.001, η^2^ = 0.127), but no effect of hemisphere (F_(1,158)_ = 3.650, *p* = 0.057, η^2^ = 0.021). We observed a group by hemisphere interaction (F_(3,158)_ = 8.879, *p* < 0.001, η^2^ = 0.144), driven by decreased right hemisphere thickness in the CON and SCZ groups relative to BD groups.

**Fig. 3:**
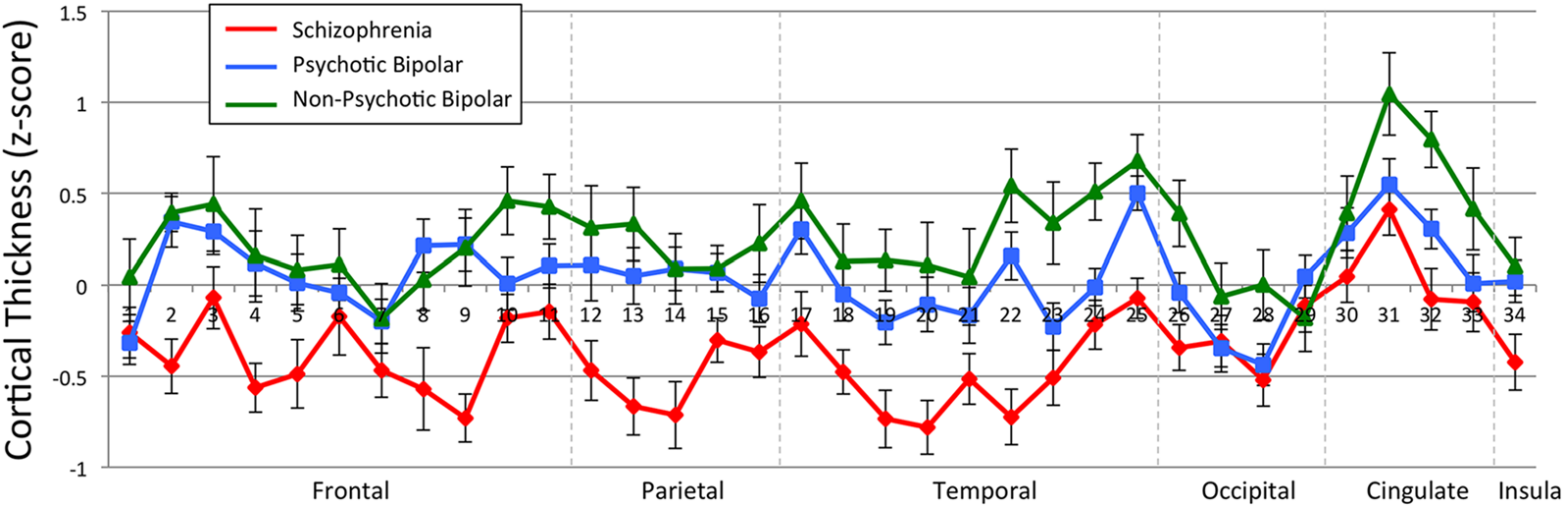
Z-scored, average cortical thickness values per region of interest. Region of interest labels correspond to those identified in Table 2. Z-scores were computed by using the mean and standard deviation of the control group as reference, and as such the healthy controls lie on the horizontal axis. Error bars represent within-group standard error

Further tests of cortical thickness averaged values across hemisphere to produce 34 cortical regions of interest to reduce the number of tests performed, given the lack of a main effect of hemisphere. Table 2 shows the results of 34 univariate tests, one for each region. After Bonferroni correction, significant effect of diagnosis was observed for the superior frontal cortex, paracentral cortex, the fusiform gyrus, as well as the caudal anterior and posterior portions of the cingulate. For visualization purposes, mean, z-scored cortical thickness values of the 34 cortical regions are depicted in Fig. 3.

**Table 2:** Estimated marginal means

	ROI	CON	SCZ	PBD	NPBD	F_(3,156)_ (p value)
Frontal	1. Frontal pole	2.736	2.675	2.650	2.696	1.279 (0.284)
	2. Superior fontal	*2.697*	*2.645*	*2.723*	*2.753*	*6.281* (*< 0.001*)
	3. Rostral middle frontal	2.284	2.302	2.320	2.355	2.239 (0.062)
	4. Caudal middle frontal	2.569	2.516	2.564	2.577	3.091 (0.029)
	5. Pars opercularis	2.612	2.590	2.599	2.645	1.660 (0.178)
	6. Pars triangularis	2.483	2.456	2.454	2.447	0.353 (0.787)
	7. Pars orbitalis	2.654	2.607	2.601	2.618	1.254 (0.292)
	8. Precentral	2.447	2.529	2.591	2.558	4.490 (0.005)
	9. Paracentral	*2.431*	*2.369*	*2.464*	*2.458*	*7.764* (*< 0.001*)
	10. Lateral orbitofrontal	2.537	2.519	2.503	2.584	2.274 (0.082)
	11. Medial orbitofrontal	2.327	2.308	2.330	2.395	2.019 (0.114)
Parietal	12. Superior parietal	2.276	2.209	2.225	2.259	2.153 (0.096)
	13. Inferior parietal	2.516	2.437	2.488	2.526	5.136 (0.002)
	14. Supramarginal	2.585	2.522	2.569	2.575	4.586 (0.004)
	15. Postcentral	2.110	2.107	2.089	2.074	0.512 (0.675)
	16. Precuneus	2.447	2.388	2.395	2.455	2.569 (0.062)
Temporal	17. Temporal pole	3.670	3.618	3.751	3.810	3.095 (0.029)
	18. Superior temporal	2.868	2.817	2.827	2.854	2.676 (0.049)
	19. Middle temporal	2.937	2.839	2.867	2.909	3.841 (0.011)
	20. Inferior temporal	2.819	2.710	2.756	2.767	4.029 (0.009)
	21. Bank Sup. Temp. Sulcus	2.627	2.566	2.542	2.568	1.438 (0.234)
	22. Fusiform	*2.744*	*2.663*	*2.726*	*2.778*	*7.476* (*< 0.001*)
	23. Transverse temporal	2.520	2.488	2.462	2.494	2.351 (0.074)
	24. Parahippocampal	2.808	2.718	2.770	2.795	2.740 (0.045)
	25. Entorhinal	3.428	3.359	3.534	3.521	3.486 (0.017)
Occipital	26. Lateral occipital	2.242	2.171	2.184	2.193	2.274 (0.082)
	27. Lingual	2.097	2.053	2.019	2.030	0.852 (0.467)
	28. Cuneus	1.928	1.889	1.878	1.917	1.799 (0.150)
	29. Pericalcarine	1.725	1.703	1.734	1.618	0.446 (0.721)
Cingulate	30. Rostral Ant. cingulate	2.776	2.777	2.851	2.856	2.501 (0.062)
	31. Caudal Ant. cingulate	*2.525*	*2.554*	*2.560*	*2.568*	*6.441* (*< 0.001*)
	32. Posterior cingulate	*2.508*	*2.468*	*2.499*	*2.615*	*7.062* (*< 0.001*)
	33. Isthmus of cingulate	2.439	2.409	2.419	2.482	1.321 (0.270)
Insula	34. Insula	3.050	3.001	3.054	3.049	3.858 (0.011)

Values indicate estimated means of subject groups, controlled for age, sex, race and total intracranial volume. Significance values are uncorrected for multiple comparisons. Italicized regions were significant after Bonferroni correcting for 34 comparisons

*ROI* region of interest, *CON* control, *SCZ* schizophrenia, *PBD* psychotic bipolar disorder, *NPBD* non-psychotic, bipolar disorder, *Temp.* temporal, *Sup.* superior, *Ant.* anterior

Results of post hoc analyses, comparing cortical thickness between individual groups in regions that showed an omnibus group effect (prior to Bonferroni correction), are shown in Table 3. After Bonferroni correction, pairwise comparisons showed cortical thinning in SCZ participants relative to other groups, most notably in specific frontal, parietal and temporal regions. However, the thickness of the anterior cingulate cortex was increased in SCZ, PBD, and NPBD participants compared to CON participants. Cortical thickness in the posterior cingulate was larger in NPBD compared to CON participants. There were no other significant cortical thickness abnormalities in either PBD or NPBD compared to CON.

**Table 3:** Pair-wise cortical thickness comparisons by diagnostic group

	ROI	CON vs. SCZ	CON vs. PBD	CON vs. NPBD	SCZ vs. PBD	SCZ vs. NPBD
Frontal	2. Superior fontal				PBD > SCZ *p *= 0.001	NPBD > SCZ *p *= 0.004
	4. Caud. Mid. frontal				PBD > SCZ *p *= 0.033	
	8. Precentral				PBD > SCZ *p *= 0.003	
	9. Paracentral	CON > SCZ *p *= 0.004			PBD > SCZ *p *< 0.001	NPBD > SCZ *p *= 0.004
Parietal	13. Inferior parietal	CON > SCZ *p *= 0.033			PBD > SCZ *p *= 0.035	NPBD > SCZ *p *= 0.003
	14. Supramarginal	CON > SCZ *p *= 0.015			PBD > SCZ *p *= 0.009	
Temporal	17. Temporal pole					
	18. Sup. temporal					
	19. Mid. temporal	CON > SCZ *p *= 0.027				NPBD > SCZ *p *= 0.032
	22. Fusiform	CON > SCZ *p *= 0.032			PBD > SCZ *p *= 0.004	NPBD > SCZ *p *< 0.001
	24. Parahippocampal					NPBD > SCZ *p *= 0.042
	25. Entorhinal					
Cingulate	31. Caud. Ant. cingulate	*SCZ > CON* *p = 0.028*	*PBD > CON* *p = 0.005*	*NPBD > CON* *p < 0.001*		
	32. Post. cingulate			*NPBD > CON* *p < 0.001*		NPBD > SCZ *p *< 0.001
Insula	34. Insula					NPBD > SCZ *p *= 0.016

Values shown reflect *p* values Bonferroni corrected for multiple comparisons for five comparisons within each region. Italicized values indicate regions that are smaller in control participants

Blank cells indicate a *p* value greater than 0.05 after correcting for multiple comparisons. No differences in PBD and NPBD groups was observed

*ROI* region of interest, *CON* control, *SCZ* schizophrenia, *PBD* psychotic bipolar disorder, *NPBD* non-psychotic bipolar disorder, *Ant.* anterior, *Sup.* superior, *Post.* posterior, *Caud.* caudal, *Mid.* middle

### Clinical relationships of cortical thickness

We performed linear regressions to predict SAPS and SANS scores based on cortical thickness, diagnostic group membership, and covariates (age, sex, and ICV). All regression models significantly predicted SAPS scores (F’s_(7,157)_ > 11.601, *p’s* < 0.001) and a diagnosis of SCZ (*p*’s < 0.001) or bipolar disorder with psychotic symptoms (*p*’s < 0.005) predicted higher SAPS. The partial correlations associated with the cortical thickness of each region, as well as the significance of those parameter estimates is shown in Table 4. After Bonferroni correction for multiple (34) tests, two regions showed significant correlations with SAPS: the middle temporal gyrus (r = − 0.257) and the fusiform area (r = − 0.255). Several additional regions were significant prior to correction for multiple comparisons including frontal (pars triangularis, lateral orbitofrontal), parietal (inferior parietal, supramarginal, precuneus) and temporal (inferior temporal, bank of the superior temporal sulcus, entorhinal) areas.

**Table 4:** Partial correlations predicting SAPS scores

	ROI	Partial correlation	*p* value
Frontal	1. Frontal pole	0.045	0.569
	2. Superior fontal	− 0.074	0.352
	3. Rostral middle frontal	− 0.026	0.748
	4. Caudal middle frontal	− 0.120	0.133
	5. Pars opercularis	− 0.141	0.075
	6. Pars triangularis	− *0.175*	*0.027*
	7. Pars orbitalis	− 0.167	0.036
	8. Precentral	− 0.117	0.142
	9. Paracentral	− 0.114	0.154
	10. Lateral orbitofrontal	− *0.163*	*0.040*
	11. Medial orbitofrontal	− 0.088	0.269
Parietal	12. Superior parietal	− 0.090	0.260
	13. Inferior Parietal	− *0.207*	*0.009*
	14. Supramarginal	− *0.172*	*0.030*
	15. Postcentral	− 0.056	0.483
	16. Precuneus	− *0.188*	*0.018*
Temporal	17. Temporal pole	− 0.099	0.217
	18. Superior temporal	− 0.107	0.179
	19. Middle temporal	*− * ***0.257***	***0.001***
	20. Inferior temporal	− *0.246*	*0.002*
	21. Bank Sup. Temp. sulcus	− *0.217*	*0.006*
	22. Fusiform	*− * ***0.255***	***0.001***
	23. Transverse temporal	0.040	0.614
	24. Parahippocampal	− 0.043	0.591
	25. Entorhinal	− *0.157*	*0.048*
Occipital	26. Lateral occipital	− 0.154	0.053
	27. Lingual	− 0.111	0.162
	28. Cuneus	− 0.037	0.643
	29. Pericalcarine	− 0.016	0.845
Cingulate	30. Rostral Ant. cingulate	0.028	0.730
	31. Caudal Ant. cingulate	0.074	0.356
	32. Posterior cingulate	− 0.115	0.151
	33. Isthmus of cingulate	0.012	0.882
Insula	34. Insula	− 0.065	0.417

Values derived from linear regression predicting SAPS scores. Significance values are uncorrected for multiple comparisons. Italicized regions indicate significance prior to multiple comparisons correction. Bolditalic regions were significant after Bonferroni correcting for 34 comparisons

*ROI* region of interest, *CON* control, *SCZ* schizophrenia, *PBD* psychotic bipolar disorder, *NPBD* non-psychotic, bipolar disorder, *Temp.* temporal, *Sup.* superior, *Ant.* anterior

All regression models also significantly predicted SANS scores (F’s_(7,157)_ > 23.780, *p’s* < 0.001), with SCZ (*p’*s < 0.001) or psychotic BD (*p*’s < 0.005) predicting higher SANS scores. After correcting for multiple comparisons, no region’s cortical thickness predicted negative symptom scores. Partial correlations prior to multiple comparisons correction are shown in Table 5. Regions that were significant prior to multiple comparisons correction included frontal (middle frontal gyrus), parietal (inferior parietal), and temporal (inferior temporal, fusiform) regions.

**Table 5:** Partial correlations predicting SANS scores

	ROI	Partial correlation	*p*-value
Frontal	1. Frontal pole	0.131	0.099
	2. Superior fontal	0.081	0.308
	3. Rostral middle frontal	*0.191*	*0.016*
	4. Caudal middle frontal	− 0.014	0.863
	5. Pars opercularis	− 0.009	0.915
	6. Pars triangularis	0.067	0.399
	7. Pars orbitalis	− 0.061	0.442
	8. Precentral	− 0.012	0.880
	9. Paracentral	0.084	0.295
	10. Lateral orbitofrontal	− 0.085	0.286
	11. Medial orbitofrontal	0.096	0.229
Parietal	12. Superior parietal	− 0.047	0.557
	13. Inferior parietal	− *0.161*	*0.043*
	14. Supramarginal	− 0.059	0.457
	15. Postcentral	0.014	0.863
	16. Precuneus	0.001	0.994
Temporal	17. Temporal pole	− 0.085	0.286
	18. Superior temporal	− 0.019	0.816
	19. Middle temporal	− 0.108	0.174
	20. Inferior temporal	− *0.142*	*0.073*
	21. Bank Sup. Temp. sulcus	− 0.083	0.296
	22. Fusiform	− *0.217*	*0.006*
	23. Transverse temporal		
	24. Parahippocampal	− 0.021	0.789
	25. Entorhinal	− 0.067	0.398
Occipital	26. Lateral occipital	− 0.114	0.154
	27. Lingual	− 0.005	0.946
	28. Cuneus	− 0.042	0.599
	29. Pericalcarine	0.038	0.631
Cingulate	30. Rostral Ant. cingulate	0.05	0.533
	31. Caudal Ant. cingulate	− 0.012	0.882
	32. Posterior cingulate	0.055	0.491
	33. Isthmus of cingulate	− 0.02	0.802
Insula	34. Insula	0.05	0.532

Values derived from linear regression predicting SANS scores. Significance values are uncorrected for multiple comparisons. Italicized regions indicate significance prior to multiple comparisons correction. No regions were significant after Bonferroni correcting for 34 comparisons

*ROI* region of interest, *CON* control, *SCZ* schizophrenia, *PBD* psychotic bipolar disorder, *NPBD* non-psychotic, bipolar disorder, *Temp.* temporal, *Sup.* superior, *Ant.* anterior

## Discussion

Our study investigated cortical thickness in young adult patients with schizophrenia and relatively homogenous clinical subgroups of bipolar I disorder patients, comprising of psychotic—(PBD) and non-psychotic (NPBD) patients with hyperthymic mania histories. We found that SCZ participants had significant cortical thinning relative to CON and BD most significantly in the frontal (i.e. paracentral), parietal (i.e. inferior parietal, supramarginal), and temporal (i.e. middle temporal, fusiform) cortices. Our results are consistent with that of most other studies which found cortical thinning in SCZ relative to control populations across a wide range of cortical regions, usually including parts of the frontal and temporal cortex (van Erp et al. 2017; Rimol et al. 2012; Sugihara et al. 2017; Knochel et al. 2016; Besteher et al. 2016; Nenadic et al. 2015; Goldman et al. 2009). A recent meta-analysis of 4474 SCZ subjects found that the largest effect sizes for lower cortical thickness compared to controls were present in the fusiform, inferior frontal (pars opercularis), lateral orbitofrontal, temporal (inferior/medial/superior), parahippocampal, insula, isthmus cingulate, and the posterior cingulate cortices (van Erp et al. 2017). Despite some inconsistencies in the specific affected regions across schizophrenia studies, which likely reflects the biological heterogeneity of patients and methodological differences, cortical thinning is considered a key pathology underlying schizophrenia. Interestingly, we also found increased cortical thickness in an isolated region—the anterior cingulate cortex (ACC)—in the schizophrenia group, as well as in both groups of bipolar disorder subjects. The ACC is a brain region critical for integrating cognitive and emotional functions in support of adaptive, goal-directed behavior (Devinsky et al. 1995). Increased ACC thickness in our subjects do not appear to be driven primarily by their relatively young age, as ACC thinning has also been seen in youth at clinically high risk for developing schizophrenia (Jung et al. 2011; Yucel et al. 2003). These findings in our patient groups differ from other studies that found gray matter reduction in the ACC in schizophrenia (Fornito et al. 2009a; Bouras et al. 2001; Hanford et al. 2016). However, increased ACC thickness has been reported in male first episode psychosis in bipolar disorder (Fornito et al. 2009b). These authors suggested that hypertrophy of regions critical for regulating the HPA axis, including the ACC, amygdala and pituitary gland, in early psychosis may be associated with an elevated stress response, which ultimately results in volumetric contraction with chronic illness. A few other authors also found increased ACC thickness in either first-episode (Adler et al. 2007) or chronic (Adler et al. 2005; Bearden et al. 2007) bipolar disorder, which was also been attributable to lithium effects in one study (Bearden et al. 2007) but not others (Fornito et al. 2009; Adler et al. 2007). Taken together, these findings suggest that while widespread cortical thinning is pathognomonic of schizophrenia, increased ACC thickness in some schizophrenia cohorts may reflect a hyperfunctional abnormality that produces an excessive stress response.

Contrary to our hypothesis, we did not find similar cortical thinning in PBD as we found in schizophrenia. Cortical thickness in both PBD and NPBD were comparable into controls, other than the increased ACC discussed above. It is notable that several other regions were larger in bipolar patients than in controls, albeit non-significantly. While these findings differ from those from a recent systematic review of bipolar disorder which reported cortical thinning in bipolar disorder (Hanford et al. 2016), normal cortical thickness have also been reported (Rimol et al. 2010, 2012). The ENIGMA Consortium conducted the largest cortical analysis of bipolar disorder patients, involving 6503 subjects and found bilateral frontal, temporal and parietal cortical thinning, especially in the left pars opercularis, fusiform gyrus and rostral middle frontal cortex (Hibar et al. 2018). When cortical thinning is observed in bipolar disorder, it is however not as pronounced as it is in schizophrenia and sometimes involves different brain regions (Knochel et al. 2016). Cortical thinning found in some bipolar disorder studies but not others appears to be due to differences in the populations studied. For example, older age and illness chronicity would be expected to lead to greater cortical thinning in bipolar disorder patients. Our study involved bipolar disorder subjects who on average, were in their mid-twenties, while most other bipolar studies include older populations. The ENIGMA bipolar study included older patients (grand mean age across sites was 37.5 years) found notably greater cortical thinning when comparing older BD patients to controls, than when younger bipolar patients were compared to controls (Hibar et al. 2018). Thus, cortical thinning may be a later presentation of bipolar disorder, which would not be evident in younger adults. Our bipolar disorder population also consisted of a more homogenous clinical cohort than those in most other studies, including only bipolar 1 disorder patients with hyperthymic manic histories. Although not directly investigated, it is plausible that bipolar disorder with hyperthymic mania may be associated with less cortical thinning than with irritable mania. Future studies comparing bipolar disorder patients based on their manic presentation, may clarify their relationship to brain structure. Finally, differences in cortical findings across studies may be related to differences in the medication and recreational drug use of the patient cohorts. Psychotropic medications used in bipolar disorder have been associated with both increased cortical thickness (e.g. lithium) (Giakoumatos et al. 2015) and cortical thinning (e.g. typical antipsychotics) (van Haren et al. 2011). Similarly, recreational substances can also exacerbate cortical thinning in patients (Hartberg et al. 2018). Our study design excluded those with a significant substance use comorbidity (i.e. substance use disorder in last 6 months), and therefore may have represented a healthier cohort with less cortical abnormality.

We did not find any notable differences between our clinical subgroups of bipolar disorder subjects. Cortical and subcortical volumetric deficits have been reported in psychotic compared to nonpsychotic bipolar disorder (Strasser et al. 2005; Altamura et al. 2017). However, consistent with our study, the ENIGMA group did not find a relationship of psychosis history and cortical thickness in bipolar subjects, although they reported an association of psychosis with surface area in isolated brain regions (Hibar et al. 2018). Thus, cortical surface or volume, which were not investigated in our study, may be more sensitive in discriminating these clinical subtypes of bipolar disorder. PBD has been reported to represent a clinical construct more closely related to schizophrenia than NPBD, based on clinical presentation, familial and genetic overlap (Moskvina et al. 2009; Ivleva et al. 2008, 2010; Green et al. 2005; Potash 2006; Tamminga et al. 2013). PBD patients also show greater cognitive dysfunction compared to NPBD, approximating that seen in schizophrenia (Martinez-Aran et al. 2008; Simonsen et al. 2011; Bourne et al. 2013).

Our results showed that the cortical thickness of several brain regions correlated with measures of positive and negative symptoms, although only relationships between temporal regions and positive symptoms survived multiple comparisons correction. The observed correlations however may have been minimized by confounders, which were not adequately corrected. For example, the recorded SAPS and SANS scores reflect symptom severity within the previous 2 weeks, and often fluctuate over time. Additionally, dimensional ratings for depression were not collected for the current study. Considering that the period between symptom assessment and brain scanning varied across patients and were in some cases lengthy (median: 14 days; mean: 19 days), clinical symptoms during scanning may not be properly captured by our instruments. In the future, studying associations with clinical measures of chronic symptoms severity, such as the Washington Early Recognition Affectivity and Psychosis (WERCAP) Screen (Hsieh et al. 2016; Mamah et al. 2014), may be more accurate in uncovering brain-behavior relationships.

Numerous factors may contribute to cortical thinning, including changes in the number, density and arrangement of cells in the cortical sheet (Hatton et al. 2013; Goldman-Rakic 1988; Kuperberg et al. 2003; Rakic and Caviness 1995). Post-mortem examination of SCZ have shown reduced neuronal size and neuropil (Rimol et al. 2012; Tandon et al. 2008; Harrison 1999). Others have reported decreases in glial density and proliferation, indicating a relative lack of gliosis (Tandon et al. 2008; Harrison 1999; Phillips et al. 2008) as well as an increase in synaptic apoptosis leading to reduced synaptic integrity in pyramidal neurons (Glantz and Lewis 2000). Decreased glial density, reduced neuronal size, and synaptic degeneration have also been observed in BD to a lesser extent than in SCZ (Tandon et al. 2008; Harrison 2002). Most of these changes are believed to be neurodevelopmental in origin (Harrison 1999, 2002; Phillips et al. 2008; Rapoport et al. 2005; Lewis and Levitt 2002) supported by reports of cortical thinning in medication naïve subjects (Narr et al. 2005a, b; Lyoo et al. 2006), as well as prior to disease onset (Ellison-Wright and Bullmore 2010; Tandon et al. 2008; Harrison 1999) and in unaffected relatives (Goghari et al. 2007).

There are some limitations to our study. Firstly, medications can influence cortical thickness, which could disproportionately affect SCZ and BD participants. Considering our sample size and the complexity of medications and doses, our current study was not adequately designed to decipher medication related effects. Typical antipsychotics, such as haloperidol, have been found to cause regional cortical thinning across wide ranges of cortical regions including frontal, temporal, and parietal areas (Dazzan et al. 2005; Lieberman et al. 2005; Navari and Dazzan 2009), in addition to increased basal ganglia volumes (Dazzan et al. 2005; Smieskova et al. 2009). Atypical antipsychotics have been associated with more muted cortical thinning compared to typical antipsychotics (Navari and Dazzan 2009; Mamah et al. 2012), and antidepressants (Duman and Monteggia 2006; Lim et al. 2012) and lithium (Bearden et al. 2007; Hafeman et al. 2012) have neurotrophic effects, which are believed to underlie symptomatic improvement. Secondly, our study results may also have been influenced by gender disparity across groups. Our subjects consisted of more males than females in the SCZ group, and more females in the BD groups. Thicker cortices have been found in regionally in women compared to men in some (Sowell et al. 2007; Koolschijn and Crone 2013; Savic and Arver 2014) but not all (Raznahan et al. 2011) studies. Sex related differences have also been reported in cortical surface area, as well as neuronal and synaptic density (Rabinowicz et al. 1999; Luders et al. 2004; Alonso-Nanclares et al. 2008). While our statistical design, treating gender as a covariate, may reduce some of this potential confound, matching studies by gender would lead to more accurate results. Finally, our study was not well powered to investigate group differences in each individual brain region. In order to reduce the number of tests performed when examining relationships between cortical thickness and symptom measures, we collapsed across hemisphere. While cortical thickness in most regions correlated across hemispheres, a group by hemisphere interaction was observed. Thus, group differences in regional cortical thickness in our patients may be specific to individual hemispheres.

## Conclusions

In conclusion, our studies investigated cortical thickness in a relatively homogenous groups of young adult schizophrenia patients and bipolar I disorder patients with hyperthymic manic histories. Regions of decreased cortical thickness were observed in schizophrenia patients, but not in either psychotic or non-psychotic bipolar patients. Thickness of the anterior cingulate cortex was increased in all patient groups. Clinical and demographic heterogeneity in patient populations may explain inconsistent findings across studies.

## Abbreviations

B-SNIP: Bipolar–Schizophrenia Network on Intermediate Phenotypes
BD: bipolar disorder
CON: healthy controls
ICV: intracranial volume
GM: gray matter
MRI: magnetic resonance imaging
NPBD: bipolar disorder with no psychotic features
PBD: bipolar disorder with psychotic features
ROI: region of interest
SANS: Scale for the Assessment of Negative Symptoms
SAPS: Scale for the Assessment of Positive Symptoms
SCID-I: Structured Clinical Interview for DSM-IV Axis I Disorders
SCID-II: Structured Clinical Interview for DSM-IV Axis II Disorders
SCZ: schizophrenia
WM: white matter
YMRS: Young Mania Rating Scale
